# Epidemiology and Pattern of Resistance of Gram-Negative Bacteria Isolated from Blood Samples in Hospitalized Patients: A Single Center Retrospective Analysis from Southern Italy

**DOI:** 10.3390/antibiotics10111402

**Published:** 2021-11-16

**Authors:** Paola Di Carlo, Nicola Serra, Sofia Lo Sauro, Vincenza Maria Carelli, Maurizio Giarratana, Juan Camilo Signorello, Alessandro Lucchesi, Giuseppe Manta, Maria Santa Napolitano, Teresa Rea, Antonio Cascio, Consolato Maria Sergi, Anna Giammanco, Teresa Fasciana

**Affiliations:** 1 Department of Health Promotion, Maternal-Childhood, Internal Medicine of Excellence G. D’Alessandro, University of Palermo, 90127 Palermo, Italy; paola.dicarlo@unipa.it (P.D.C.); mariasanta.napolitano@unipa.it (M.S.N.); antonio.cascio03@unipa.it (A.C.); anna.giammanco@unipa.it (A.G.); 2 Department of Public Health, University Federico II of Naples, 80131 Napoli, Italy; nicola.serra@unina.it (N.S.); teresa.rea@unina.it (T.R.); 3 Emergency Unit, Umberto I Hospital, 94100 Enna, Italy; Sofia.lo.sauro95@gmail.com; 4 Microbiology Unit, Sant’Elia Hospital, 93100 Caltanissetta, Italy; enza.carelli@alice.it (V.M.C.); mgiarratana@alice.it (M.G.); 5 Hypatia School of Medicine (UNIPA-Caltanissetta), University of Palermo, 90127 Palermo, Italy; juancamilo.signorello@community.unipa.it; 6 Hematology Unit, Istituto Scientifico Romagnolo per lo Studio e la Cura dei Tumori (IRST) IRCCS, 47014 Meldola, Italy; alessandro.lucchesi@irst.emr.it; 7 Intensive Cure Unit (ICU), Sant’Elia Hospital, 93100 Caltanissetta, Italy; giuseppemantanest@gmail.com; 8 Children’s Hospital of Eastern Ontario, University of Ottawa, Ottawa, ON K1H 8L1, Canada; bioteclab@gmail.com

**Keywords:** bacteria, survival time, MDR, infection

## Abstract

Background: Blood culturing remains the mainstream tool to inform an appropriate treatment in hospital-acquired bloodstream infections and to diagnose any bacteremia. Methods: A retrospective investigation on the prevalence of Gram-negative bacteria (GNB) and their resistance in hospitalized patients by age, sex, and units from blood cultures (BCs) was conducted from January 2018 to April 2020 at Sant’Elia hospital, Caltanissetta, southern Italy. We divided the patient age range into four equal intervals. Results: Multivariate demographic and microbiological variables did not show an association between bacteria distributions and gender and age. The distribution by units showed a higher prevalence of *Klebsiella pneumoniae* and *Acinetobacter baumannii* in the intensive care unit (ICU) and *Escherichia coli* in the non-intensive care units (non-ICUs). The analysis of antibiotic resistance showed that *E. coli* was susceptible to a large class of antibiotics such as carbapenem and trimethoprim-sulfamethoxazole. *K. pneumoniae* showed a significant susceptibility to colistin, tigecycline, and trimethoprim-sulfamethoxazole. From the survival analysis, patients with *E. coli* had a higher survival rate. Conclusions: The authors stress the importance of the implementation of large community-level programs to prevent *E. coli* bacteremia. *K. pneumoniae* and *E. coli* susceptibility patterns to antibiotics, including in the prescription patterns of general practitioners, suggest that the local surveillance and implementation of educational programs remain essential measures to slow down the spread of resistance and, consequently, increase the antibiotic lifespan.

## 1. Introduction

The diagnostic and therapeutic implications of the role of blood culturing in hospital settings continue to raise much debate with a growing number of articles comparing rapid molecular diagnostic tests and blood culturing [1,2,3,4,5,6]. Both older and more recent studies have strengthened knowledge regarding the beneficial impact of blood culturing on patient care in critical patients in intensive care units (ICUs) [7,8,9] as well as in patients with other underlying disorders [10]. Collecting blood culture data can help survey bacterial trends in the ICU and/or non-intensive care units (non-ICUs) and contribute to assessing the epidemiology of antimicrobial resistance [11,12]. Recently, the role of blood culturing was highlighted by a review of the timing of blood sample collections, especially in hospital settings [7]. Furthermore, an Italian study published in 2020 on bloodstream infections confirmed the higher prevalence of Gram-negative bacteria (GNB) compared with Gram-positive bacteria (GPB), with *E. coli* and *K. pneumoniae* dominating among GNB [8].

The pattern of GNB can show different geographic distributions as well as their antibiotic susceptibility. In our geographical area, we recently described a new *K. pneumoniae* clone outbreak and the isolation of *E. coli* species in endocarditis and biliary disorders [11,12,13,14,15]. Antimicrobial resistance (AMR) is one of the biggest problems in global public health. Despite attempts to control the spread of these infections at a local and national level, the epidemiological situation for carbapenem-resistant *K. pneumoniae* (CRKp) is a significant concern in the Mediterranean area [11,12].

In 2017, Italy adopted its first national action plan on antimicrobial resistance 2017–2020, which works through the synergy between national, regional, and local data [16,17]. As part of the regional action plan regarding nosocomial infections and the prevention of antimicrobial resistance, the Sicilian health department has implemented a surveillance system on resistance rates (RRs) in hospitals [17].

Thus, the purpose of this study was to evaluate the current prevalence of GNB and their resistance pattern in hospitalized patients by age, sex, and units through the analysis of the blood cultures of patients admitted to Sant’Elia hospital in Caltanissetta in the middle of Sicily.

## 2. Materials and Methods

This study was a retrospective investigation of blood culture (BC) samples positive for GNB detected in all adult (≥18 years old) patients hospitalized for 48 h at the Sant’Elia Hospital of Caltanissetta from January 2018 to April 2020.

The samples for the blood cultures were collected aseptically by peripheral venipuncture from patients with a suspected bloodstream infection according to the CDC guidelines [18].

Each positive BC result was assessed for contamination (false-positive) and, according to established criteria, the false-positive isolates were excluded from the study [19].

The records of patients included age, sex, isolated organisms, hospital ward, and antibiotic susceptibility patterns. The data records were obtained from the database using institutional electronic microbiological information. Bacterial identification and antimicrobial susceptibility testing were carried out using either the Phoenix Automated Microbiology System (Becton Dickinson Diagnostic Systems, Sparks, United States) or the Vitek-2 System (BioMérieux, Marcy l’Etoile, France).

According to the European Committee on Antimicrobial Susceptibility Testing (EUCAST) breakpoints, the antimicrobial sensitivity test of the strains was determined as previously reported [14,15,20]. A routine surveillance measure for infection control was applied at the Sant’Elia Hospital, Caltanissetta, Italy, as part of the GISIO and SPIN UTI Italian surveillance projects [21,22].

According to EUCAST, K. pneumoniae and P. aeruginosa are naturally resistant to ampicillin, and A. baumannii and P. aeruginosa to ertapenem.

The local Ethics Committee approved the study as part of a thesis during a student medical course at the School of Medicine, University of Palermo, Italy. Unfortunately, considering the retrospective microbiological nature of the study, we could not obtain the consent of patients to use their demographic data. We allotted arbitrary numbers to all isolates assigned to the study to guarantee their anonymity.

## 3. Statistical Analysis

Data are presented as numbers and percentages for categorical variables, and continuous data are expressed as the mean ± standard deviation (SD) or as the median and interquartile range (IQR).

The multiple comparison chi-squared test was used to define the significant differences in the percentages for the unpaired data. If the chi-squared test was positive (*p*-value less than 0.05), then a post-hoc Z-test was performed to locate the highest or lowest significant presence.

The test used for a normal distribution the Shapiro–Wilk test.

The t-test was used to test the differences between two means of paired/unpaired data. Alternative non-parametric tests were used when the distribution was not expected. The Mann–Whitney test was used to compare two independent samples.

A survival analysis was performed considering the more frequent isolates and compared with the log-rank test. Kaplan–Meier graphs were generated where a positive BC > 48 h after admittance better fit the definition of infection as hospital-acquired.

All tests with *p* < 0.05 were considered significant. The statistical analysis was performed by Matlab statistical toolbox version 2008 (MathWorks, Natick, MA, USA) for Windows at 32 bit.

## 4. Results

During the study period, we collected 336 positive blood cultures. Of those, 105 were positive for GNB; 65.71% (69/105) were isolated from males and 34.29% from females (36/105).

The age range was 26–91 years with a mean of 67.85 years and a standard deviation of 15.71 years.

In Table 1 we report the strains isolated in our sample, stratified for gender to evaluate the relationship between the isolates and gender.

Notably, no significant association between males and females in terms of isolates was observed (*p* = 0.44). We observed that, among the isolates in the male group, the most frequent Gram-negative strains were *K. pneumoniae* (49.3%, 118 *p* < 0.0001) and *A. baumannii* (20.3%, *p* = 0.012) whereas in the female group, the most frequent Gram-negative strains were *K. pneumoniae* (38.9%, *p* < 0.0001) and *E. coli* (27.8%, *p* = 0.0019).

To individualize the possible relationship between isolates and age, we divided the age range into four equal intervals. We considered the quartile values for this scope as Q1 = 58, Q2 = 71, and Q3 = 79. Table 2 shows no significant difference for any isolate with regard to age.

In the analysis of the age intervals, *K. pneumonia* was the most frequent strain identified in all age groups (42.3%, 44.0%, 46.2%, and 50.0%; *p* < 0.0001). To analyze the relationship between the strains and hospital wards (HWs), we divided the data into two groups: patients hospitalized in non-intensive care units (Cardiology, Vascular Surgery, Surgery, Hematology, Hemodialysis, Long-Term Care, Infectious Disease, Medicine, Neurosurgery, Oncology, Emergency Unit, Pneumology, and Urology) and patients hospitalized in the ICU (Table 3).

We found that *K. pneumoniae* and *E. coli* were the most frequent isolates in the non-ICU cases (21.0%, *p* < 0.0001; 17.1%, *p* < 0.0001, respectively) whereas, in ICU patients, the most frequent isolates were *K. pneumoniae* (24.8%, *p* < 0.0001) and *A. baumannii* (14.3%, *p* < 0.0001).

In addition, as shown in Table 3, we observed that *A. baumannii* was most frequent in ICU patients compared with non-ICU patients (78.9% > 21.1%, *p* = 0.0192) whereas *E. coli* was most frequent in non-ICU cases compared with those in the ICU (90% > 10%, *p* = 0.0004).

In Table 4, we report the percentages of antibiotic resistance for every isolate as well as the statistical analysis of the antibiogram results and GNB.

In the penicillin class, we found a statistically significant resistance to amoxicillin clavulanic acid (*p* = 0.0135) and piperacillin tazobactam (*p* = 0.0002).

*E. coli* was the isolate most susceptible to amoxicillin clavulanic acid (36.8%, *p* = 0.0013) and piperacillin tazobactam (20%, *p* = 0.001). However, *E. cloacae, P. mirabilis*, and *S. marcescens* were significantly susceptible to amoxicillin clavulanic acid (*p* = 0.0091, *p* = 0.0279, and *p* = 0.0173, respectively).

Regarding the cephalosporin class, all cephalosporin generations tested (second, third, and fourth) showed a significant susceptibility pattern with the isolates. *E. coli* and *S. marcescens* showed a susceptibility to all generations of cephalosporins whereas *K. pneumoniae* was significantly resistant to cefepime (76.6%, *p* = 0.0292).

In the fluoroquinolone class, several GNBs such as *Citrobacter freundii* (0.0%, *p* = 0.0031), *Enterobacter aerogenes* (33.3%, *p* = 0.0417), *Enterobacter cloacae* (0.0%, *p* = 0.001), *Proteus mirabilis* (0.0%, *p* = 0.0081), and *Serratia marcescens* (0.0%, *p* = 0.0031) were susceptible.

For the aminoglycoside class, *A. baumannii* was the isolate most resistant to gentamicin (88.9%, *p* = 0.023), amikacin (83.3%, *p* = 0.023), and fosfomycin (100%, *p* = 0.0054). Regarding the carbapenem class, *A. baumannii* was resistant to ertapenem (83.3% *p* = 0.0299) and meropenem (100%, *p* = 0.0261) whereas *E. coli* showed a significant susceptibility to all carbapenem drugs tested (imipenem: 0.0%, *p* = 0.0015; ertapenem: 5.0% *p* = 0.0005; meropenem: 0.0%, *p* = 0.0014). Among the other rare GNB strains detected, *S. marcescens* isolates showed a significant resistance to colistin (100%, *p* = 0.0145).

As shown in Table 5, we evaluated the relationship between the single strains and their resistance or susceptibility to the antibiotics tested by a multivariate analysis.

*K. pneumoniae* was more susceptible to colistin (15.9%, *p* < 0.0001), fosfomycin (46.8%, *p* = 0.0238), trimethoprim-sulfamethoxazole (23.4%, *p* < 0.0001), and tigecycline (12.8%, *p* < 0.0001). *A. baumannii* (19/105 isolates) was the strain that showed a major susceptibility to colistin (6.7%, *p* < 0.0001) and tigecycline (0.0%, *p* = 0.0002). *E. coli* (20/105 isolates) was more susceptible to amikacin (0.0%, *p* = 0.0105), ertapenem (5%, *p* = 0.0416), imipenem (0.0%, *p* = 0.0318), and meropenem (0.0%, *p* = 0.0105). In addition, a survival analysis was performed with these bacteria. For this, the more frequently identified bacteria, *E. cloacae* and *E. aerogenes*, were grouped as *Enterobacter* spp. All less frequently occurring bacteria such as *Klebsiella oxytoca* (#2), *S. marcescens* (#2), *P. mirabilis* (#1), and *C. freundii* (#2), were grouped as Others. We observed no significant difference in the survival curves (log-rank test, *p* = 0.44). The mean survival time and survival rate for patients with *A. baumannii*, *Enterobacter* spp., *E. coli*, *K. pneumoniae*, Others, and *P. aeruginosa* were 65.4 days and 52.6% = 10/19 pts, 49.7 days and 50% = 3/6 pts, 51.9 days and 95% = 19/20 pts, 81.6 days and 54.2% = 26/48 pts, 40.8 days and 71.4% = 5/7 pts, and 27.4 days and 40% = 2/5 pts, respectively.

In addition, we analyzed the survival rates and compared them, considering every GNB. A significant difference in the survival rates was observed (*p* = 0.0248). Notably, according to a post-hoc Z-test, patients with *E. coli* had a higher survival rate than other GNBs (95%, *p* = 0.0032).

## 5. Discussion

The authors considered only blood cultures collected from patients hospitalized for 48 h because BC collection can reflect the circulation of hospital germs. We aimed to obtain BCs uncomplicated by bacteremia but showing a bloodstream infection. Moreover, we advised the physicians to be careful when ordering BCs, especially in the first three days after admission, and to avoid antibiotic administration.

Our data showed that the most frequently isolated pathogens were *K. pneumoniae*, *A. baumannii,* and *E. coli,* as in other Italian studies [8,12,16].

In our geographic area, these Gram-negative strains have been reported as being responsible for hospital-acquired infections in ICUs, biliary samples of patients with biliary and pancreatic disorders, and in endocarditis patients [13,14,15,16,17,18,19,20,21,22,23].

Focusing on age, we found that *K. pneumonia* was more frequent in every group analyzed. This pathogen is endemic in European countries bordering the Mediterranean Sea, including the region of Sicily. *K. pneumoniae* is well-known to clinicians as a cause of nosocomial infections, especially ICU bloodstream infections, and is associated with a high 90 d mortality [24].

Although we did not find any significant differences based on gender in our sample, we observed that *E. coli* bacteremia was more frequent in women; the female anatomy and vaginal colonization by *E. coli* can be considered to be risk factors for *E. coli* bacteremia from a urinary tract infection (UTI).

Although the statistical analysis by age did not show a significant difference in the different age groups, the frequency analysis showed a higher prevalence of *K. pneumoniae* and *E. coli* in patients older than 79 years. Both were the most common causative agents of an ICU admission in the geriatric population.

These results confirmed the substantial burden of these pathogens in older adults, justifying the implementation of community-level programs to prevent Gram-negative bacteremia and ICU admission in this age group [21,22,23,24,25,26,27].

The isolates from non-ICU and ICU settings showed that *E. coli* was prevalent in non-ICU settings, as reported in other Italian studies and high-income countries [27,28]. Urogenital infections accounted for more than half of all *E. coli* bacteremia episodes; in hospitalized individuals, in-dwelling vascular and urinary catheters increased the risk of *E. coli* bacteremia. In our geographical area, this pathogen has been isolated in poultry food and hospital settings as well as in the bile microbiome of patients with cancer [14,15,21,26,29].

The survival analysis of our study of the hospital mortality rate showed that the hospital *E. coli* bacteremia mortality was lower than bacteremia due to other strains. This follows a recent review where the case fatality rate for in-hospital *E. coli* bacteremia did not differ appreciably from that in the general population [30].

Regarding antimicrobial resistance, this study showed that, as the pressure of antibiotics has been less relevant in this area of Sicily, *K. pneumoniae* and *A. baumannii* remain sensitive to colistin and tigecycline. Of interest were the findings that several isolates of *K. pneumoniae* showed a susceptibility to trimethoprim-sulfamethoxazole.

The susceptibility to folate pathway inhibitors was somewhat surprising and was linked to the low abuse of this drug in both inpatients and outpatients. 

Other studies have reported this treatment option and its use in combined therapy against *K. pneumoniae* [31]. 

These data suggest XDR Gram-negative bacteria but not MDR Gram-negative bacteria increase mortality due to BSI [32].

In our study, the antimicrobial data analysis showed that *E. coli* was susceptible to carbapenem, aminoglycoside, fosfomycin, and trimethoprim-sulfamethoxazole. This was consistent with other Italian studies that have shown that *E. coli* resistance to the carbapenem class and other antibiotic families is decreasing [28,33]. We took into account fosfomycin resistance patterns because, in our study, we collected blood samples. Recently, the literature has shown how intravenous fosfomycin may play a role in the association with other antibiotics in the treatment of bloodstream infections due to MDR-GNB [34,35].

Regarding the statistical analysis of the susceptibility to different antibiotic classes of the GNB strains isolated in our hospitalized patients, colistin showed the lowest resistance pattern to GNB isolated in our blood culture samples, especially for strains isolated in the ICU such as *A. baumannii*. However, in 2019, Agodi et al. collected Sicilian antimicrobial data showing that resistance to colistin increased over three years [32]. Our study collected data through microbiological records, therefore, we did not investigate the clinical records on sequential therapy with other antibiotics. However, our team—including ICU clinicians—could clarify that, according to the susceptibility pattern of all *A. baumannii* isolated in the blood samples, all ICU patients in this study received targeted regimens, especially intravenous monotherapy colistin.

This study suggests that the prevalence of antibiotic resistance is higher in metropolitan cities; if we analyzed the surveillance data for hospitals in the provinces, the findings may be different. This study highlights the need for every clinician and infectious disease specialist to know the epidemiological data of the hospital where they work. Clinicians must be required to provide a punctual and monthly verification by the agency of the European Union (https://www.ecdc.europa.eu/en/publications-data/surveillance-antimicrobial-resistance-europe-2019 (accessed on 1 September 2021) and also local antibiotic resistance data collection agencies.

This methodology, assessing the antibiotic resistance model by geographical area, must also be carried out in a timely manner in an ongoing capacity because climate change and climate catastrophes could impact the management of infectious diseases in both inpatients and outpatients [36].

The recent pandemic has alerted us to the need for a greater territorial management outside hospitals, especially of the elderly with mental alterations and comorbidities [37].

## 6. Conclusions

This study elucidated the prevalence and antibiotic resistance pattern of GNB in the Mediterranean area and confirmed the substantial burden of *K. pneumoniae* and *E. coli* bacteremia in southern Italy. The prevalence of *E. coli* in non-ICU settings, especially in females and the elderly, indicated that the implementation of large community-level programs to prevent Gram-negative bacteremia in ICUs should be considered once again.

Surveillance and epidemiological studies help clinicians fit the magnitude of antimicrobial resistance and establish early measures to slow down the spread of resistance, consequently increasing the antibiotic lifespan.

## 7. Limits of the Study

This study was subject to the strict regulation of antibiotics and well-established antibiotic stewardship in the S.Elia Caltanissetta Hospital. Therefore, we could not determine the effect of inpatient antibiotic administration on the outcome of BCs drawn in all units, including in the ICU. In addition, data were collected from a sample obtained from a single hospital in southern Italy; therefore, the findings must be interpreted with caution and further studies should be conducted on a larger sample involving several hospitals from different geographical areas.

## Figures and Tables

**Table 1:** Relationship between 105 strains isolated in blood culture and gender.

	Total (#105)	Males (#69)	Females (#36)	Males vs. Females
				*p*-Value (Test Type)
**Age**				
Mean ± SD	66.59 ± 15.64	65.54 ± 15.66	68.61 ± 15.63	rN, *p* < 0.0001 (SW)
Median (IQR)	71 (58–79)	70 (55–79)	73 (62–80)	0.30 (MW)
**Strain isolates**	**% (#)**	**% (#)**	**% (#)**	
(1) *K. pneumoniae*	45.7 (48)	49.3 (34)	38.9 (14)	
(2) *E. coli*	19.0 (20)	14.5 (10)	27.8 (10)	
(3) *A. baumannii*	18.1 (19)	20.3(14)	13.9 (5)	
(4) *P. aeruginosa*	4.8 (5)	2.9 (2)	8.3 (3)	
(5) *E. aerogenes*	2.9 (3)	1.4 (1)	5.6 (2)	*p* = 0.44 (Cm)
(6) *E. cloacae*	2.9 (3)	2.9 (2)	2.8 (1)	
(7) *C. freundii*	1.9 (2)	2.9 (2)	0.0 (0)	
(8) *K. oxytoca*	1.9 (2)	2.9 (2)	0.0 (0)	
(9) *S. marcescens*	1.9 (2)	1.4 (1)	2.8 (1)	
(10) *P. mirabilis*	0.9 (1)	1.4 (1)	0.0 (0)	
		*p* < 0.0001 * (Cm)	*p* < 0.0001 * (Cm)	
**Analysis into groups** ***p*-value (test type)**		(1) **, *p* < 0.0001 (Z) (3) **, *p* = 0.012 (Z)	(1) **, *p* < 0.0001 (Z) (2) **, *p* = 0.0019 (Z)	

* = Significant test; ** = most frequent bacteria; Cm = multicomparison chi-squared test; Z = post-hoc Z-test; SW = Shapiro–Wilk test for normal distribution; rN = reject normality; MW = Mann–Whitney test.

**Table 2:** Analysis of 105 Gram-negative bacteria (GNB) stratified by class of age.

Isolates (#)	Age Intervals			
	(24–58) (#26)	(58–71) (#24)	(71–79) (#25)	(79–90) (#30)
(1) *K. pneumoniae* (48)	42.3 (11)	45.8 (11)	48.0 (12)	46.7 (14)
(2) *E. coli* (20)	15.4 (4)	20.8 (5)	16.0 (4)	23.3 (7)
(3) *A. baumannii* (19)	19.2 (5)	20.8 (5)	24.0 (6)	10.0 (3)
(4) *P. aeruginosa* (5)	0.0 (0)	4.2 (1)	4.0 (1)	10.0 (3)
(5) *E. aerogenes* (3)	3.8 (1)	4.2 (1)	0.0 (0)	3.3 (1)
(6) *E. cloacae* (3)	7.7 (2)	0.0 (0)	4.0 (1)	0.0 (0)
(7) *K. oxytoca* (2)	3.8 (1)	4.2 (1)	0.0 (0)	0.0 (0)
(8) *S. marcescens* (2)	0.0 (0)	0.0 (0)	4.0 (1)	3.3 (1)
(9) *C. freundii* (2)	7.7 (2)	0.0 (0)	0.0 (0)	0.0 (0)
(10) *P. mirabilis* (1)	0.0 (0)	0.0 (0)	0.0 (0)	3.3 (1)
**Analysis into groups** ***p*-value (test type)**	*p* < 0.0001 * (Cm)	*p* < 0.0001 * (Cm)	*p* < 0.0001 * (Cm)	*p* < 0.0001 * (Cm)
	(1) **, *p* < 0.0001 (Z)	(1) **, *p* < 0.0001 (Z)	(1) **, *p* < 0.0001 (Z)	(1) **, *p* < 0.0001 (Z)
				(2) **, *p* = 0.0433 (Z)
				(6) ***, *p* = 0.0433 (Z)
				(7) ***, *p* = 0.0433 (Z)
				(9) ***, *p* = 0.0433 (Z)

* = Significant test; ** = most frequent bacteria; *** = less frequent bacteria; Cm = multicomparison chi-squared test; Z = post-hoc Z-test.

**Table 3:** Comparison of 105 Gram-negative bacteria (GNB) detected in non-intensive care units (non-ICUs) and the intensive care unit (ICU).

	Isolates									
Operative Units	AcB	CiF	EnA	EnC	EsC	KlO	KlP	PrM	PsA	SeM
(OU)	% (nr.)	% (nr.)	% (nr.)	% (nr.)	% (nr.)	% (nr.)	% (nr.)	% (nr.)	% (nr.)	% (nr.)
non-ICUs (n = 52)	7.7 (4/52)	0.0 (0)	1.9 (1)	1.9 (1)	34.6 (18)	1.9 (1)	42.2 (22)	1.9 (1)	3.9 (2)	3.9 (2)
Percentages defined on a single bacterium	21.1 (4/19)	0.0 (0)	33.3 (1)	33.3 (1)	90 (18)	50 (1)	45.8 (22)	100 (1)	40 (2)	100 (2)
ICU (n = 53)	28.3 (15/53)	3.8 (2)	3.8 (2)	3.8 (2)	3.8 (2)	1.9 (1)	49.1 (26)	0.0 (0)	5.7 (3)	0.0 (0)
Percentages defined on a single bacterium	78.9 (15/19)	100 (2)	66.7 (2)	66.7 (2)	10 (2)	50 (1)	54.2 (26)	0.0 (0)	60 (3)	0.0 (0)
Single bacterium										
ICU vs. non-ICUs	ICU **				non-ICUs **					
	*p* = 0.0192	*p* = 0.5	*p* = 1.0	*p* = 1.0	*p* = 0.0004	*p* = 1.0	*p* = 0.57	*p* = 1.0	*p* = 1.0	*p* = 0.5

AcB = *A. baumannii*; CiF = *C. freundii*; EnA = *E. aerogenes*; EnC = *E. cloacae*; EsC = *E. coli*; KlO = *K. oxytoca*; KlP = *k. pneumoniae*; PrM = *P. mirabilis*; PsA = *P. aeruginosa*; SeM = *S. marcescens*; * = significant test; ** = most frequent; *** = less frequent; C = multicomparison chi-squared test; Z = post-hoc Z-test.

**Table 4:** Statistical analysis on the percentages of antibiotic resistance of 105 Gram-negative isolates.

Antibiotic Category	Antibiotic	AcB	CiF	EnA	EnC	EsC	KlO	KlP	PrM	PsA	SeM	*p*-Value (Test)
		%	%	%	%	%	%	%	%	%	%	
		(#)	(#)	(#)	(#)	(#)	(#)	(#)	(#)	(#)	(#)	
	Amoxicillin clavulanic acid	/	100 (2/2)	100 (1/1)	100 (3/3)	36.8 (7/19)	100 (2/2)	80.4 (37/46)	100 (1/1)	/	100 (2/2)	*p* = 0.0135 * (C)
												EsC ***, *p* = 0.0013 (Z)
Penicillin	Ampicillin	100 (5/5)	/	/	/	100 (6/6)	/	92.9 (13/14)	/	100 (1/1)	/	*p* = 0.83 (C)
	Piperacillin tazobactam	100 (1/1)	50 (1/2)	66.7 (2/3)	0.0 (0/3)	20 (4/20)	100 (2/2)	80.4 (37/46)	0.0 (0/1)	60 (3/5)	0.0 (0/2)	*p* = 0.0002 * (C)
												EnC ***, *p* = 0.0091 (Z)
												EsC ***, *p* = 0.001 (Z)
												PrM ***, *p* = 0.0279 (Z)
												SeM ***, *p* = 0.0173 (Z)
Cephalosporin second generation	Cefoxitin	100 (7/7)	100 (2/2)	/	100 (1/1)	18.2 (2/11)	100 (1/1)	53.3 (16/30)	/	100 (1/1)	0.0 (0/1)	*p* = 0.0166 * (C)
												EsC ***, *p* = 0.0125 (Z)
												SeM ***, *p* = 0.0353 (Z)
Cephalosporin third generation	Ceftazidime	/	50 (1/2)	66.7 (2/3)	33.3 (1/3)	35 (7/20)	50 (1/2)	83.3 (40/48)	100 (1/1)	50 (2/4)	0.0 (0/2)	*p* = 0.0064 * (C)
												EsC ***, *p* = 0.0084 (Z)
												SeM ***, *p* = 0.0087 (Z)
	Cefotaxime	100 (9/9)	50 (1/2)	66.7 (2/3)	66.7 (2/3)	55 (11/20)	100 (2/2)	83.3 (40/48)	100 (1/1)	100 (2/2)	0.0 (0/2)	*p* = 0.0343 * (C)
												EsC ***, *p* = 0.0287 (Z)
												SeM ***, *p* = 0.0009 (Z)
Cephalosporin fourth generation	Cefepime	/	50 (1/2)	66.7 (2/3)	0.0 (0/3)	10 (2/20)	50 (1/2)	76.6 (36/47)	0.0 (0/1)	40 (2/5)	0.0 (0/2)	*p* < 0.0001* (C)
												KlP **, *p* = 0.0292 (Z)
												EnC ***, *p* = 0.0198 (Z)
												EsC ***, *p* = 0.0005 (Z)
												PrM ***, *p* = 0.0429 (Z)
												SeM ***, *p* = 0.0318 (Z)
Fluoroquinolone class	Ciprofloxacin	89.5 (17/19)	0.0 (0/2)	33.3 (1/3)	0.0 (0/3)	60 (12/20)	100 (1/1)	83.3 (40/48)	0.0 (0/1)	50 (2/4)	0.0 (0/2)	*p* = 0.0003* (C)
												CiF ***, *p* = 0.0031 (Z)
												EnA ***, *p* = 0.0417 (Z)
												EnC ***, *p* = 0.001 (Z)
												PrM ***, *p* = 0.0081 (Z)
												SeM ***, *p* = 0.0031 (Z)
Aminoglycoside	Fosfomycin	100 (8/8)	0.0 (0/1)	66.7 (2/3)	0.0 (0/3)	10 (2/20)	50 (1/2)	46.8 (22/47)	100 (1/1)	100 (2/2)	0.0 (0/2)	*p* = 0.0006* (C)
												AcB **, *p* = 0.0054 (Z)
												EnC ***, *p* = 0.041 (Z)
												EsC ***, *p* = 0.0051 (Z)
	Amikacin	83.3 (10/12)	0.0 (0/2)	33.3 (1/3)	0.0 (0/3)	0.0 (0/20)	50 (1/2)	60.4 (29/48)	0.0 (0/1)	50 (2/4)	0.0 (0/2)	*p* < 0.0001 * (C)
												AcB **, *p* = 0.023 (Z)
												EnC ***, *p* = 0.0363 (Z)
												EsC ***, *p* = 0.0002 (Z)
	Gentamicin	88.9 (16/18)	50 (1/2)	0.0 (0/2)	0.0 (0/3)	20 (4/20)	50 (1/2)	70.8 (34/48)	0.0 (0/1)	50 (2/4)	0.0 (0/2)	*p* = 0.0001* (C)
												AcB **, *p* = 0.023 (Z)
												EnA ***, *p* = 0.0206 (Z)
												EnC ***, *p* = 0.0113 (Z)
												EsC ***, *p* = 0.0014 (Z)
												PrM ***, *p* = 0.0318 (Z)
												SeM ***, *p* = 0.0206 (Z)
Carbapenems	Imipenem	80 (8/10)	0.0 (0/1)	50 (1/2)	0.0 (0/1)	0.0 (0/12)	/	68.4 (13/19)	/	100 (1/1)	0.0 (0/1)	*p* = 0.0024 * (C)
												EsC ***, *p* = 0.0015 (Z)
	Meropenem	83.3 (15/18)	50 (1/2)	66.7 (2/3)	0.0 (0/3)	0.0 (0/20)	50 (1/2)	70.8 (34/48)	0.0 (0/1)	25 (1/4)	0.0 (0/2)	*p* < 0.0001* (C)
												AcB **, *p* = 0.0299 (Z)
												EnC ***, *p* = 0.0182 (Z)
												EsC ***, *p* < 0.0001 (Z)
												PrM ***, *p* = 0.0413 (Z)
												SeM ***, *p* = 0.0299 (Z)
	Ertapenem	100 (7/7)	0.0 (0/1)	50 (1/2)	0.0 (0/3)	5 (1/20)	100 (2/2)	66.7 (22/33)	100 (1/1)	100 (1/1)	0.0 (0/2)	*p* < 0.0001* (C)
												AcB **, *p* = 0.0261 (Z)
												EnC ***, *p* = 0.0268 (Z)
												EsC ***, *p* = 0.0005 (Z)
												SeM ***, *p* = 0.040 (Z)
Folate pathway inhibitors	Trimethoprim-sulfamethoxazole	88.2 (15/17)	50 (1/2)	50 (1/2)	0.0 (0/2)	35 (7/20)	0.0 (0/2)	23.4 (11/47)	0.0 (0/1)	100 (2/2)	0.0 (0/2)	*p* = 0.0004* (C)
												AcB **, *p* = 0.0003 (Z)
Glycylcycline class	Tigecycline	0.0 (0/2)	0.0 (0/2)	/	0.0 (0/1)	5.6 (1/18)	0.0 (0/2)	12.8 (5/39)	100 (1/1)	100 (2/2)	/	*p* = 0.0032* (C)
												PsA **, *p* = 0.0119 (Z)
Polymyxin class	Colistin	6.7 (1/15)	0.0 (0/2)	0.0 (0/1)	0.0 (0/2)	5.6 (1/18)	0.0 (0/2)	15.9 (7/44)	100 (1/1)	20 (1/5)	100 (2/2)	*p* = 0.0109* (C) SeM **, *p* = 0.0145 (Z)

AcB = *A. baumannii*; CiF = *C. freundii*; EnA = *E. aerogenes*; EnC = *E. cloacae*; EsC = *E. coli*; KlO = *K. oxytoca*; KlP = *k. pneumoniae*; PrM = *P. mirabilis*; PsA = *P. aeruginosa*; SeM = *S. marcescens*; * = significant test; ** = most frequent resistant bacteria; *** less frequent resistant bacteria; C = multicomparison chi-squared test; Z = post-hoc Z-test.

**Table 5:** Resistance and susceptibility of 105 Gram-negative bacteria.

***A. baumannii***
*p* < 0.0001 * (C)
Colistin ***, *p* < 0.0001 (Z)
Tigecycline ***, *p* = 0.0002 (Z)
***C. freundii***
*p* = 0.52 (C)
***E. aerogenes***
*p* = 0.92 (C)
***E. cloacae***
*p* = 0.0094 * (C)
Amoxicillin
clavulanic acid **, *p* = 0.0039 (Z)
***E. coli***
*p* < 0.0001 *(C)
Ampicillin **, *p* < 0.0001 (Z)
Cefotaxime **, *p* = 0.0015 (Z)
Ciprofloxacin **, *p* = 0.0002 (Z)
Amikacin ***, *p* = 0.0105 (Z)
Imipenem ***, *p* = 0.0318 (Z)
Meropenem ***, *p* = 0.0105 (Z)
Ertapenem ***, *p* = 0.0416 (Z)
***K. oxytoca***
*p* = 0.33 (C)
***K. pneumoniae***
*p* < 0.0001* (C)
Amoxicillin clavulanic acid **, *p* = 0.021 (Z)
Ampicillin **, *p* = 0.0391 (Z)
Piperacillin tazobactam **, *p* = 0.021 (Z)
Cefotaxime **, *p* = 0.0058 (Z)
Ceftazidime **, *p* = 0.0058 (Z)
Ciprofloxacin **, *p* = 0.0058 (Z)
Colistin ***, *p* < 0.0001 (Z)
Fosfomycin ***, *p* = 0.0238 (Z)
Tigecycline ***, *p* < 0.0001 (Z)
Trimethoprim-sulfamethoxazole ***, *p* < 0.0001 (Z)
***P. mirabilis***
*p* = 0.37 (C)
***P. aeruginosa***
*p* = 0.49 (C)
***S. marcescens***
*p* = 0.0142 * (C)
Amoxicillin clavulanic acid **, *p* = 0.0181 (Z)
Colistin **, *p* = 0.0181 (Z)

* = Significant test; ** = most frequent bacteria; C = multicomparison chi-squared test; Z = post-hoc Z-test; ** antibiotic more resistant; *** antibiotic more susceptible.
